# A Journey in Capacity Building: Revisiting the Mullins Framework for Meaningfully Engaging Patients in Patient Centered Outcomes Research

**DOI:** 10.3389/fpubh.2018.00343

**Published:** 2018-12-03

**Authors:** M. Paige Powell, Amanda J. Young, Hyunmin Kim

**Affiliations:** ^1^Division of Health Systems Management and Policy, University of Memphis School of Public Health, Memphis, TN, United States; ^2^Department of Communication and Film, College of Communication and Fine Arts, Affiliate Faculty, School of Public Health, University of Memphis, Memphis, TN, United States

**Keywords:** patient engagement, patient centered outcomes research, mullins framework, hard-to-reach patients, cancer disparities

## Abstract

This paper reviews the implementation of the UNITE for Better Health Outcomes Eugene Washington PCORI Engagement Award project using Mullins and colleagues' Framework for Meaningfully Engaging Patients in Patient Centered Outcomes Research (PCOR) and the advantages and disadvantages of this framework. We combine Mullins' framework with the ten themes for guiding future studies in PCOR also developed by Mullins' research group. We interviewed patient stakeholders at the end of the engagement award and include patient stakeholder perspectives of how well we performed each of these steps. Despite some breakdowns in trust, which were eventually repaired, we successfully identified patient and family stakeholders; built partnerships with patients, researchers, providers, and community groups; explained the purpose of our project and the importance of PCOR; developed training materials for patients and providers; and updated our key constituents throughout the process. Overall, we believe combining Mullins' framework with the ten themes provides a solid roadmap for implementing a PCORI engagement award. Our main challenge was recruiting and keeping hard-to-reach patients and caregivers involved in the project. We believe this was due to our limitations more so than the framework. Based on the lessons we learned, we provide concrete recommendations for others who want to engage hard-to-reach patients using the Mullins framework.

## Background

UNITE for Better Health Outcomes grew out of concern among researchers and providers about significant and growing health disparities in cancer mortality between white and African Americans in Memphis, Tennessee. The city of Memphis is predominantly African American (63.6%). Memphis is one of the poorest cities in the US, with a per capita income of $22,728, and over a quarter of its residents live in poverty (1). In a study conducted by Sinai Urban Health Institute, the Metropolitan Chicago Breast Cancer Task Force and Avon Foundation Cancer Crusade, Memphis was identified as the U.S. city with the largest disparity in breast cancer mortality rates between non-Hispanic Black and non-Hispanic White women (2). Although the incidence rate is approximately the same for non-African American and White women, African American women are almost two times more likely to die from breast cancer than White women (1.95 mortality rate ratio). Similar disparities exist for prostate cancer (2.74 mortality rate ratio), colon and rectal cancer (1.71 mortality rate ratio), and lung and bronchus (1.22 mortality rate ratio) (3). Recognizing the high prevalence and mortality rate of cancers among African Americans in its service areas, the goal of this project was to build on our growing capacity to engage cancer patients in PCOR, aiming to eventually improve cancer-related comparative effectiveness research and cancer-related health outcomes of minority and hard-to-reach cancer patients in Memphis. We discovered a lack of both patient engagement and patient-centered efforts to address these disparities, and applied and received a Eugene Washington PCORI Engagement Award to build the community and connections that would be required to conduct patient centered outcomes research (PCOR) and to develop training materials for patients, providers and researchers to work together.

The specific aims of this project were to (1) Create a Patient-Stakeholder Council (PSC) composed of cancer patients, caregivers, family members, clinicians, researchers, and social service providers to serve on a committee responsible for steering patient engagement in PCOR in Shelby County; (2) form Patient-Caregiver Groups (PCGs) that included four groups of seven to 10 patients, caregivers, and family members that engaged hard-to-reach patients and fleshed out patients' priorities for PCOR, and (3) to have the PSC become the voice for patients' needs and ideas for improving the healthcare system as it relates to cancer outcomes and research. Although this project was not designed to improve the health or functioning of the community, it laid the ground work to eventually do so with patients as partners in the research process. The researchers involved in this project entered with no specific expectation of the community's needs, but used the engagement and development process to identify those.

We developed our community strategy using the framework developed by Mullins and colleagues at the University of Maryland for engaging hard-to-reach patients (4). They pioneered much of the evidence base for engaging hard-to-reach patients in the research process and in developing PCOR capacity in minority patient groups. The steps for Building and Maintaining Trust include Identifying, Partnering, Explaining, Doing, and Updating. Although this process is shown linearly, the authors also include a feedback loop. We learned that this arc did not only occur from the beginning of the project until the end but was a continuous process that occurred through the project period and beyond.

In their research article published in the *Journal of Comparative Effectiveness Research*, Mullins's team developed guidance for “how and where to engage study participants and how to keep them engaged” (5). They provided ten “Themes for Guiding Future PCOR Studies” that we incorporated into their framework for maintaining and building trust (5). The purpose of this paper is to describe the implementation of these themes as a framework in the context of building an initial engagement group and to discuss the advantages and disadvantages of our application of this model.

## Methods

The University of Memphis IRB determined that the Unite for Better Health Outcomes project fell under quality improvement and was therefore non-human subjects research. Toward the end of the project, the researchers sought IRB approval to conduct key informant interviews with patients and family caregivers who had been involved in the project from the beginning. These participants completed informed consent and were interviewed either in person or over the phone using an IRB approved protocol. We applied quotes and stories obtained through these key informant interviews to supplement our perspectives about the advantages and disadvantages of using the Mullins framework to build engagement in research.

## Building PCOR capacity with cancer patients in memphis

### Identifying and partnering (steps 1 – 4)

#### (Step 1) bring PCOR to where people live and (step 2) use a period of pre-engagement when recruiting research participants and partners

We first created a Patient Stakeholder Council (PSC): a group of former cancer patients or family caregivers who would work with us as co-researchers. We began the journey by visiting local neighborhoods where cancer mortality rates were much higher for African Americans than whites. Through our partnership with Methodist Le Bonheur Healthcare (MLH), we were able to leverage the services of several of their staff who are cancer navigators. These individuals found locations in their neighborhoods to recruit our PSC members. The informational sessions held in these locations were our period of pre-engagement. During each informational session, our PSC leaders, two patients who helped develop the engagement award proposal, spoke about their cancer journeys and the importance of patients getting involved in improving the quality of cancer care. The project lead (author 1) would then speak briefly about what PCORI does and the goals of our project. The attendees filled out an information sheet and marked their preferred level of participation: monthly, 2–3 times per year, or email/newsletter updates. Those who indicated monthly were chosen for the council based on gender, type of cancer, and neighborhood in which they lived. Those who were not selected and those who indicated they preferred involvement 2–3 times per year were later contacted about participating in focus groups in their neighborhoods. However, part of the feedback that we received was that all participants wanted a fuller understanding of what we were trying to accomplish. According to one PSC member,

“…I've read bits and bits of the grant. But I was never given a total full grant. And I don't know whether that was supposed to be like that. But I think to get the full value from a person, the person should know the full value of the grant, what the grant is all about. Not bits and pieces. And I think that's what we were given, bits and pieces.”

#### (Step 3) involve the full-spectrum of people that will be affected by the research, including hard-to-reach patients

We discovered during the project that we had particular difficulty in recruiting hard-to-reach patients—patients with tenuous community connections, lower socioeconomic status, and limited access to health care. We brainstormed ways to reach these potential participants but had very little success. Those the PSC considered truly hard-to-reach attended two of the 16 focus groups, with a total of seven participants. Yet many of the participants indicated that word was “trickling” down through communication from those participants to others in their families and neighborhoods, giving us hope that in a future PCOR project, we will have more success in engaging this population. According to one PSC member, one of the barriers to success was a lack of training:

“I think the person (sic) that we recruited were good people. I think the recruitment is good…but they need some training. You just can't take a person who's not been exposed to community work, this level of interaction. You can't expect him to do a perfect job.”

A second PSC member identified another recruitment barrier:

“I learned, that when you putting people together, you've got to let them know that if we talking about black people, you've got to talk about the less fortunate black folks. Not those as well-connected. And I think it was easy to get folks who's already connected, already were connected, you know? But it takes a little difficulty to get folks that [are] the less fortunate.”

#### (Step 4) build and maintain trust for active patient engagement

We worked consistently throughout the project to maintain the relationship of trust that we established with the PSC leaders and members of the PSC. As with any workgroup, we had moments that required some repair. About halfway through the first phase of the project, the leadership team, which included the PSC leaders, made a decision about how to recruit people from neighborhoods where the struggle with poverty was more severe. When we talked about the targeted neighborhood with the larger group, a different plan emerged. Later one of the PSC leaders told us that he felt he had been “thrown under the bus.” He is an elderly African American man who has spent years working among people struggling to feed their families, let alone access adequate health care. At the time, he did not question the decision, and we were unaware of his feelings until much later. During our interview he said, “everybody had their reason for doing certain things” and expressed that the experience did not lessen his regard for the project. It did, however, hurt his feelings at the time.

The other PSC leader, however, saw this as a breach of trust. Having talked in the small leadership meeting about targeting a specific neighborhood, and then seeing that decision overturned in the larger group, she felt that she and her co-leader were left out of the decision-making process. Later that week, all on the leadership team discussed the issue, and it became clear that our communication was flawed. We decided that the two leaders would talk with the larger group at the next meeting and would take the lead on group discussions in future meetings.

Later, both PSC leaders described the healing process. One said, “I think we've learned a lot from maybe some good moments and maybe some not so good moments that we can move forward and do better.” In the end, she said that the most important result from participating in the project was that she had found her voice. The other PSC member noted, “I guess overall, I guess what I'm saying, that I think I've been a benefit to PCORI. But on the other hand, PCORI's been a benefit to me.”

### Explaining (steps 5 and 6)

#### (Step 5) provide education on what is meant by the term “research”

During the pre-engagement period, we spoke with communities about PCORI's mission and what types of “research” we conducted and how it differed from clinical research. Given the level of mistrust in the community, we were very careful in the beginning to not use the word “research” but to explain the work so that we didn't immediately turn people away. Once we formed the PSC, we gave the members more information about PCORI. We also had them brainstorm ways to talk with their communities about research. During interviews following the project, PSC members were asked, “Has it [the project] changed the way you view medical research?” One of the members responded,

“Definitely, it has. I had not put a lot of thought into medical research. I know it was there. I know it was happening. I know we had to have it….But now, after my experience with PCORI, I'm more conscious. I'm more willing to participate in research in all levels. Not only in the cancer research, but all levels of medical research. So it has definitely made me more conscious.”

Yet, we still had struggles with our PSC members often talking about the provision of care when we tried to solicit information about research. In fact, in spite of our prompts, our first round of focus groups continually skewed away from research and into care.

We regrouped with our PSC to discuss this issue and learned quickly that the majority of the PSC members were confused about the project. In fact, many of the professionals on the project had also slipped into a mind frame of patient/provider interactions rather than participant/researcher. While acknowledging that a person's physician is often both a clinician and a researcher, our overall goal was to address the relationship between medical researchers and the community. To explore that, we asked PSC members to take a moment and write down three words that came to their minds in response to the term “medical research.” We were dismayed to hear the words when we asked participants to call them out. “Tuskegee,” “guinea pig,” and similar negative words signified that the historical trauma of immoral research perpetrated against African Americans was still deeply engrained, even among individuals who were committed to advancing research in their communities. From that point forward, we worked to frame discussions even more specifically around research, rather than care, and to elicit and address concerns about medical research.

#### (Step 6) realize that people may not be willing to openly disclose medical information

From the first day of the project, we saw first-hand the power of story-telling. Our PSC members were open and honest about their conditions and treatment. They wanted to tell their stories and have their voices heard, and later said that telling their stories was both a significant contribution to the project and a validating experience for them. We asked permission of some participants to use details of their stories for scenarios in training sessions. Those participants readily agreed, but we also modified details so that no one could be identified.

### Doing

Once the recruited members agreed to join the PSC, we held an orientation session where our members learned more about PCORI and how to support them and encourage them to tell their own stories. We also worked on team-building and held a session with a local Toastmasters group to help our members become more comfortable with public speaking. We then met monthly and developed themes for focus groups, focus group protocols, recruitment materials and efforts, and training and development materials. Although many parts of this framework are not tied directly to themes identified by Kaufmann, we regularly revisited parts of the strategies within the first half of the framework: Identifying, Partnering, and Explaining.

At each meeting, we devoted one half-hour to fellowship and then reviewed the previous progress. They broke into small groups to work on whichever task was next (e.g., focus group protocols). We learned over time how to better elicit participation from the more reserved PSC members. One of our stakeholders found that some meetings were repetitive, as if we were “beating a dead horse,” but also found both value and areas for improvement in bringing more perspectives to the forefront. When asked what we could do to improve, she addressed the need to make sure everyone had a voice:

“[Be] able to utilize our stakeholders more so because a lot of our stakeholders…they have a lot of information to share themselves. And I think a lot of them develop because some of them came in being very quiet and then we were able to see another side, where actually they really talked. And we had some really knowledgeable stakeholders. And I think they have something to share. So I had made a suggestion to [the project lead] that going forward in some of our meetings, if we could like rotate the facilitator and allow different people to facilitate the meeting. And therefore have it more interactive.”

### Updating (steps 7–10)

#### (Step 7) keep people up-to-date on what is going on with the research and (8) provide a lay summary of study findings at an end-of-study celebration

We proposed that our PSC members would talk quarterly with their communities about the project's progress. However, many of our members felt uncomfortable doing so. The project lead attended churches and community meetings to give periodic updates, but we had hoped to empower the PSC members to do this. We invited the same people for some of the focus groups, so we provided updates at the beginning of the second and third round of focus groups. We revisited the aims of the study within the context of what we proposed and what we were able to accomplish. We gave an overview of PCORI at every meeting and included a large discussion on PCORI during the training and development sessions. After the third round of focus groups, we gave a wrap up that discussed why we asked the questions that we did, what we intended to do with the information, and how that fit into the larger framework of PCORI's principles of engagement. Finally, we held a celebration dinner that included PSC members, project staff, focus group participants and significant others. During this meeting, we reviewed the evaluation results of our training session.

#### (Step 9) make a sincere effort to “give back” to the community

Many of the researchers and project staff made efforts to give back to the community by participating in health fairs, the LIVE breast cancer summit, and other community efforts. The project lead attended church with many of the PSC members to talk about what the project was doing. However, many of our PSC members also realized that they could use this project to give back to their own communities:

“I guess my greatest challenge, I think we've already touched on it, is making the others realize that people who are less fortunate have not, don't have the same privileges, don't have the same access, because of their own lacking or because people just don't reach out to them as much as they should. I think that effort should be double in order to reach [them].”

#### (Step 10) recognize that people make healthcare choices and participate in research based upon who they are as individual persons, not just as patients

As researchers, we learned a great deal about attitudes and decision making about both decisions to seek treatment and participate in research. An inherent mistrust of medicine and medical research persists in the African American community. People also mentioned fatalistic attitudes, spirituality, and other factors that led some people to participate and others to decline. Because we continually had challenges with our PSC members and participants reverting to the provider/treatment relationship, there were more discussions about that than participation in research. Our PSC members also realized that people see things differently when it comes to cancer treatment decisions.

“Well I learned to be a bit more sensitive about cancer, its effect, both physically and mentally on patients. I think I've learned to appreciate that better. The other thing I've learned, how to deal with the other people who have not been touched, or not been associated with anyone who has cancer, how to deal with them to make them more sensitive, and make them more conscious of the journey of people who have cancer.”

Overall, we performed well with the beginning steps of “Identifying and Partnering,” but had some challenges on recruiting the truly “hard-to-reach” groups and stumbled on a couple of occasions with building and maintaining trust. We learned that we needed to improve the “Explain” step by helping our team members better understand the project and its goals, particularly with regard to research and how this project was related to research efforts. Our PSC members gave us recommendations on how to improve “Doing” the project and how to give back to the community more in the “Updating” phase.

## Discussion

Many of the difficulties our project faced and lessons the team learned relate to communication and organization, particularly with recruiting, giving all participants the “full picture” of the project, and sharing leadership. One of these problems involved recruiting the full spectrum of people who would be affected by our training and research, especially the poorest of the poor (Mullins, Step 3). As white researchers, we often talk about “the African American community” as if it were monolithic. We learned that there is a disconnect between specific communities and that significant barriers exist between them. One member of the PSC had specialized training and years of experience in reaching out to those in Memphis in the deepest poverty. In an interview, he expressed that his skills were not fully utilized during the project. We recommend checking in with PSC members on a more regular basis to make sure their skills are being used and that they are comfortable with their level of participation and opportunity. We also recommend recruiting multiple members who have worked closely with and are able to communicate with hard-to-reach groups to provide a synergistic effect.

We also should have involved our Patient Partner leaders more in planning and running the various meetings. The project lead usually facilitated the meetings. Although the leaders of the PSC were involved in decision making and were asked for input in the bi-monthly meetings, we now believe it would have been more advantageous for them to run the PSC meetings. We believe that would have built more trust among participants, especially our PSC leaders. We recommend that PSC leaders receive some training in facilitation or moderation if they do not have experience, but that they also be allowed more responsibility overall. We also learned that we should debrief with the leaders following every PSC meeting because they often picked up on things that the academic members did not. Acting on their insights immediately would have helped in engaging more of the community and kept the project on track.

Additionally, we learned that our Patient Partners and Stakeholder Council members wanted and needed to know more details about the PCORI award. We gave our two main patient partners copies of the final proposal accepted by PCORI, but did not talk more about this in the PSC meetings. At the beginning of the project, we were trying to avoid using the word “research” because our Patient Partners suggested that would alienate the audience and impair recruitment. However, explaining research is a fundamental step in Mullins' framework. One of the lessons learned is that those who are building coalitions among hard-to-reach groups may want to tackle this early in the process.

An additional part of the communication challenges involved harnessing the power of “trickle-down” communication. When trying to change recruiting practices to reach the more disadvantaged groups, many of the PSC members justified their reticence by saying that the message was trickling down to others. However, that still did not provide a voice to everyone in the community, especially those we intended to reach. In hindsight, we should have paused and explored this phenomenon more. We not only need to develop a better understanding of what is trickling down, how it is being communicated, but also provide an avenue for communication to flow back up to the larger group. Despite these difficulties, we believe that the Mullins framework, and the lessons we learned while implementing it, provides an opportunity for other groups to learn from our experiences when beginning their own engagement efforts.

## Conclusions

While there are several groups that have developed conceptual models for engaging patients in research (e.g., CANCERGEN and BC SUPPORT) (6, 7), we believe that Mullins, Kaufmann, and colleagues have developed an outstanding model for not only engaging patients in research, but also in outlining an engagement process. The “Framework for Patient Engagement” combined with the “Themes for Guiding Future PCOR Studies” provided a step-by-step approach that considered multiple points along the recruitment and engagement continuum, including feedback loops that helped us revisit parts of the project as we progressed. Based on the continued engagement and feedback provided by our PSC members, combining these two models offers a roadmap for others to follow who want to build and maintain partnerships with disparate groups in their communities. Future efforts should focus on explaining the project and research better to the entire team, checking in and following up with members on a consistent basis to ensure that their skills are being utilized appropriately, empowering members of the community to lead the meetings and focus groups, and working on multiple avenues of communication to ensure that all community members' voices are heard.

Although several suggestions of practical methods for engaging hard-to-reach patients were identified by Mullins and used in our project, we still had trouble in reaching the more disadvantaged community members. We had very mixed groups, some with more advantages than others, similar to Varming and colleagues in their study of the feasibility of using patient-centered education for self-management of chronic diseases (8). As Woolf and colleagues suggest, “The long-term relationships and collaboration on which such engagement depends—whether for research, practice, or social action—requires infrastructure and an investment of resources to maintain those relationships.” (9). PCORI provided those initial resources through the Eugene Washington Engagement Award, and we now have the commitment of our members to continue on to the next stage: conducting a comparative effectiveness research study to reduce disparities and improve patient centered outcomes in cancer care.

## Ethics statement

This study was carried out in accordance with the recommendations of the University of Memphis Institutional Review Board and with written informed consent from all subjects. All subjects gave written informed consent in accordance with the Declaration of Helsinki. The protocol was approved by the University of Memphis IRB PRO-FY2017-599.

## Author contributions

All authors contributed to conception and design. MP, AY, and HK drafted manuscript. AY conducted key informant interviews. All authors read and approved the final manuscript. All authors agreed to be accountable for all aspects of the work.

### Conflict of interest statement

The authors declare that the research was conducted in the absence of any commercial or financial relationships that could be construed as a potential conflict of interest.
